# Imaging of Malignant Primitive Tumors of the Spine

**DOI:** 10.5334/jbsr.1410

**Published:** 2018-09-06

**Authors:** Meriem Mechri, Hend Riahi, Imed Sboui, Mouna Bouaziz, Filip Vanhoenacker, Mohamed Ladeb

**Affiliations:** 1 Institut Kassab of Orthopaedics, TN; 2 AZ Sint-Maarten, Mechelen and University (Hospital) Antwerp/Ghent, BE

**Keywords:** spine, bone tumors, radiograph, CT, MRI

## Abstract

Primary malignant tumors of the spine are rare and mainly include chordoma, chondrosarcoma, Ewing sarcoma or primitive neuroectodermal tumor, and osteosarcoma. The final diagnosis is based on the combination of patient age, topographic and histologic features of the tumor, and lesion pattern on computed tomography (CT) and magnetic resonance (MR) imaging. Imaging evaluation includes radiography, CT, bone scintigraphy, and MR imaging. CT is more useful than radiography for evaluating location of the lesion and analyzing bone destruction and matrix, whereas MR has unmatched ability to assess soft tissue extension. This pictorial review provides an overview of the most prevalent primitive malignant tumors of spine.

## Introduction

**Figure 1:**
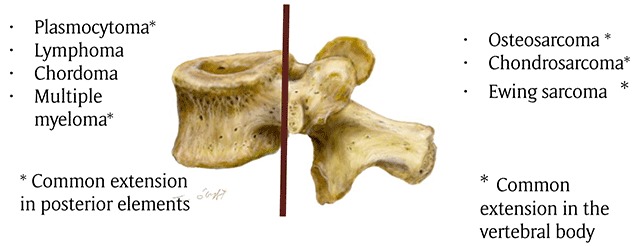
Drawing showing the common distribution of primary malignant tumors of spine. The brown line indicates the border between the vertebral body and posterior elements.

Primary malignant tumors of the spine are rare and mainly include chordoma, chondrosarcoma, Ewing sarcoma or primitive neuroectodermal tumor (PNET), and osteosarcoma [1]. In patients under 30 years of age, tumors of the spine are fairly uncommon and are generally benign, except for Ewing sarcoma and osteosarcoma [1]. Imaging evaluation of patient presenting with a vertebral tumor usually includes radiography, computed tomography (CT), bone scintigraphy and magnetic resonance (MR) imaging. CT is superior to conventional radiography for evaluating lesion location and analyzing tumor matrix and bone changes. Whole-body MR has been evaluated in various oncologic indications [2]. MR has unmatched ability to assess extension to the bone marrow, soft tissue, and spinal canal and allows evaluation of treatment response. Technetium bone scintigraphy can be helpful in revealing an osseous lesion and to document multifocality [3]. Unfortunately, bone scintigraphy is nonspecific and often unable to help distinguishing between benign tumors, tumor-like conditions, and malignant tumors. The final diagnosis of spinal tumors is based not only on patient age, histologic features, and topographic features of the tumor (Figure 1), but also on analysis of the pattern on imaging. Table 1 summarizes the demographic data and localization of most common malignant primary tumors of the spine. The purpose of this article is to review the imaging features of the most prevalent primitive malignant tumors of spine.

**Table 1:** Epidemiology of malignant primary tumors of the spine.

	Mean Age	Gender	% spinal involvement	Preferential location in spine	Preferential location in vertebrae	Involvement of adjacent vertebral level

Osteosarcoma	38 years	M>F	4% of all osteosarcoma	Thoracic and lumbar levels >sacrum and cervical	Posterior elements (79%), partial vertebral body extension	17% of cases Disk space is usually preserved
Chondrosarcoma	45 years	M>F	<12% of all chondrosarcoma	Thoracic>cervical> lumbar	Posterior elements 40%, Vertebral body, both 45%	35% of cases Disk space is usually preserved
Ewing sarcoma	19.3 years	M>F	3–10%	Sacro-coccygeal region>lumbar >thoracic>cervical spine	Posterior elements 60%	8% of cases Disk space is usually preserved
Chordoma	50–60 years	M>F		Spheno occipital skull base: 35% Sacro-coccygeal area: 50% Vertebral body: 15% Cervical spine>thoracic>lumbar	Vertebral body with sparing to the posterior elements	Soft tissue extension “mushroom appearance” spanning several segments and sparing the disks
Plasmocytoma	>60% years	F>M	25–50%	Thoracic vertebra	Vertebral body++ Extension in pedicles is frequent	May extend through the intervertebral disk
Lymphoma	40–60 years	M>F	1%–3% of all lymphomas 7% of primary bone tumors	–	Vertebral body++ Posterior involvement rare	Contiguous vertebral involvement is possible Disk space is usually preserved
Multiple myeloma	Rare under 30 years	M>F	Skeletal involvement in 80–90% of cases Vertebral involvement in 65% of cases	–	Vertebral body++ Extension in pedicles is frequent	May extend through the intervertebral disk

## Osteosarcoma

Osteosarcoma is a primitive malignant bone tumor characterized by the production of osteoid or immature bone from neoplastic cells [4]. Spinal osteosarcoma comprises 3.6–14% of all primary spinal tumors and 4% of all osteosarcomas [5]. They occur in older age groups than osteosarcoma of the appendicular skeleton (mean age of 38 years), with a male predilection. In 79% of cases, the tumor arises in the posterior elements with partial vertebral body involvement. Involvement of two vertebral levels is seen in 17% of cases [5,6]. Radiographs and CT usually show a mixed osteosclerotic-osteolytic lesion. A heterogenous soft tissue mass with ossified and non ossified components is commonly associated. Rarely, tumors with marked mineralization originating in the vertebral body may manifest as an “ivory vertebra” (sclerosing osteoblastic osteosarcoma). A purely lytic pattern is also seen in various subtypes such as telangiectatic osteosarcoma (predominant cystic architecture simulating aneurysmal bone cyst).

MR of spinal osteosarcoma is usually nonspecific. Mineralized component of the tumor shows low signal on T1-weighted images (WI) and T2-WI, whereas the nonmineralized component displays a high signal on T2-WI. Fluid-fluid levels have been described in association with telangiectatic osteosarcoma [7]. Expansive pattern cortical destruction, soft tissue extension, and pathological fractures may be seen [4].

Local recurrence is estimated at 20% after en bloc excision and 60% after locoregional excision [4,8].

## Chondrosarcoma

Chondrosarcomas represent a heterogenous group of tumors characterized by their capacity of cartilage formation [9]. Chondrosarcoma accounts for 10% of all primary bone tumors [10] and less than 12% may occur in the spine [11]. The thoracic spine is the most frequent localization (accounting for 60%), followed by the cervical and lumbar spine [12]. Spinal chordosarcoma may arise in the posterior elements (40%), in the vertebral body, or both [13]. Men are affected two or four times as much as women and the mean age of patients is 45 years [8].

**Figure 2:**
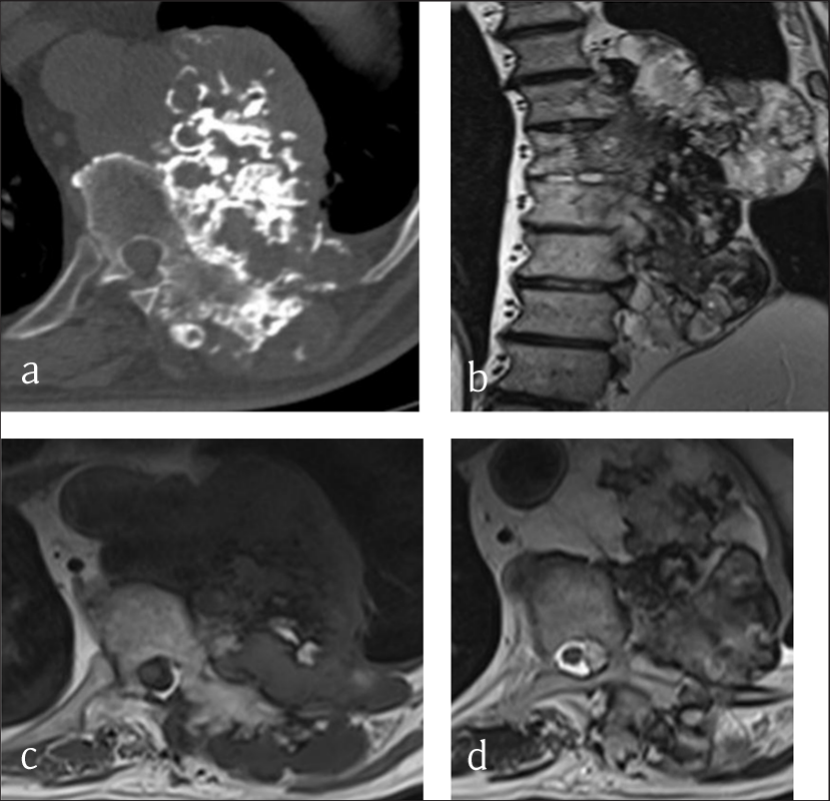
**Chondrosarcoma of T8, T9 and T10. (a)** Axial CT scan shows a large mass arising from the vertebral body with ring-and-arc calcifications. MRI **(b)** coronal T2-WI, **(c)** Axial T1-WI and **(d)** Axial T2-WI show a heterogenous mass consisting of lobules of intermediate signal intensity on T1-WI with residual intralesional bony trabeculae and high signal on T2-WI sourrounded by hypointense ring-and-arcs.

Most lesions represent primary chondrosarcoma; however, secondary chondrosarcoma may also occur when osteochondroma (solitary or multiple with hereditary multiple exostoses) undergoes malignant transformation [8]. Radiographs demonstrate a well-defined mass with internal calcifications [13]. Chondroid matrix mineralization (Figure 2a) is better demonstrated by CT, showing typical “rings and arcs” [14]. CT may demonstrate a geographic osteolysis with sclerotic borders [1]. It may also allow evaluation of paravertebral extension of the tumor, the shifting and infiltration of surrounding structures, and involvement of adjacent levels. Involvement of adjacent vertebral levels by extension through the disk is seen in approximately 35% of cases, and adjacent ribs may be affected in thoracic neoplasms [8]. Occasionally, spinal chondrosarcoma may present as a lytic lesion involving the vertebral body or compression fracture of the superior or inferior end-plates [13].

**Figure 3:**
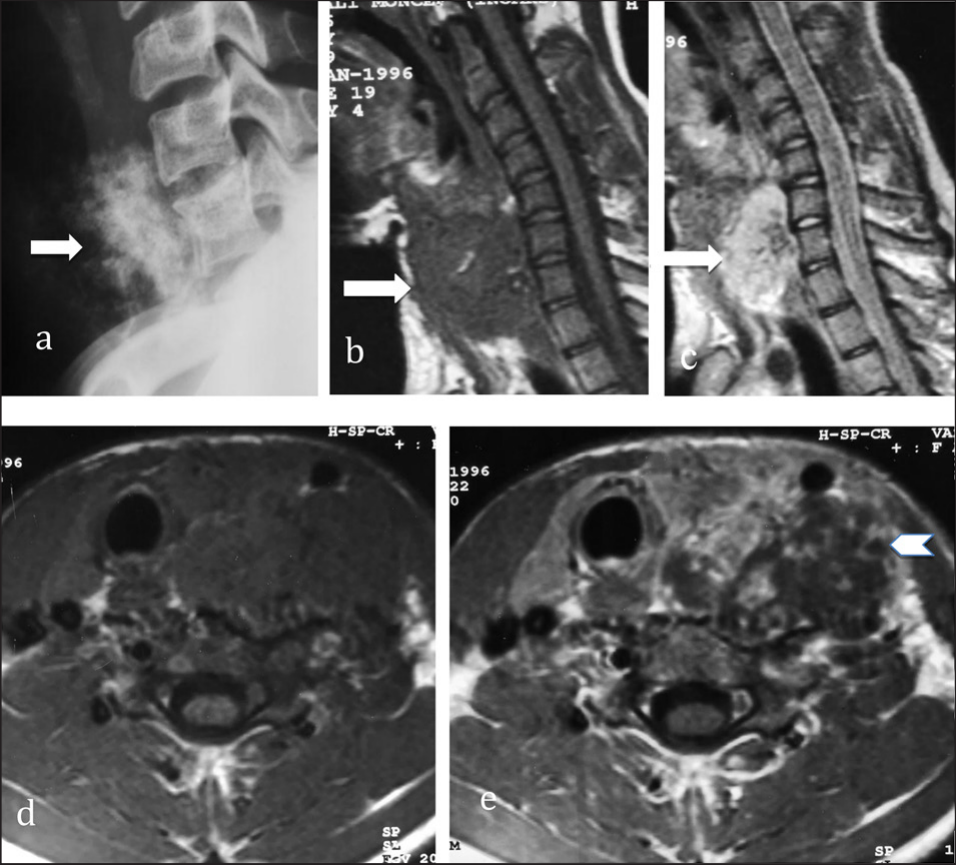
**Chondrosarcoma of C7. (a)** Lateral radiograph of cervical spine shows a large iuxta-osseous calcified mass arising from the vertebral body of C7 (arrow). **(b, c, d, e)** MR: Sagittal T1-WI, Sagittal T2-WI, Transversal T1-WI, Transversal T1-WI after gadolinium contrast injection show lobulated mass of intermediate signal intensity on T1-WI and high signal intensity on T2-WI (arrows) with ring-and-arc enhancement (arrowhead).

**Figure 4:**
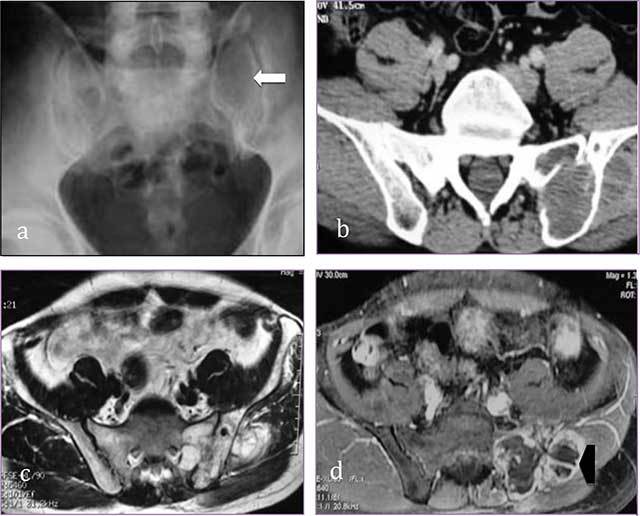
**Sacral chondrosarcoma. (a)** Plain radiograph of pelvis shows an ill-defined osteolytic lesion of left sacrum (white arrow). **(b)** transversal CT scan shows extension through the left sacroiliac joint, the ilium and the gluteal muscles. **(c, d)** MR: transversal T2-WI, transversal T1-WI after gadolinium contrast injection show a lobulated mass predominantly of high signal intensity on T2-WI with ring-and-arc enhancement (arrowhead).

On MR the tumor is of low signal on T1-WI (Figure 2c) and heterogeneous with areas of low and high signal intensities on T2-WI (Figure 2b and 2d) and Short Tau Inversion-Recovery (STIR) images, corresponding to mineralized and nonmineralized matrices. In addition, MR is superior to CT in depicting the epidural and intraforaminal extension highlighting possible compression of the neural structures. Fat-suppressed contrast-enhanced T1-WI show peripheral and lobulated rim enhancement (Figures 3e and 4d). Chondrosarcoma tends to recur if inadequately treated. En bloc resection provides the best chance of survival and the lowest rate of local recurrence [15].

## Chordoma

Chordoma is a rare malignant neoplasm arising from the embryonic remnants of the primitive notochord and accounts for 2–4% of all primary malignant bone neoplasm, with an estimated prevalence of 0.51 per million. Chordomas generally occur in middle-aged patients, with a peak prevalence in the 5th–6th decades. Spinal chordomas have a 2:1 male–female ratio [16].

Eighty-five per cent of chordomas occur in spheno-occipital skull base (35%), sacro-coccygeal area (50%) [17], and the vertebral bodies (15%). Spinal chordomas arise more frequently in the cervical spine than in the thoracic and lumbar regions [17]. This tumor may present a significant variability in histology and is divided into three types: conventional, chondroid, and dedifferentiated [18]. Clinical manifestation is often subtle because chordomas are slow-growing lesions.

Radiographs and CT scan usually show a lytic lesion of a vertebral body associated with a soft-tissue mass with a “mushroom” appearance, spanning several segments and sparing the disks. Areas of amorphous calcifications are noted in 40% of chordomas of the mobile spine and in up to 90% of sacrococcygeal lesions [14]. Bone sequestra may be also seen. In some cases sclerosis predominates, leading to an “ivory” vertebral appearance, but this pattern is rare.

**Figure 5:**
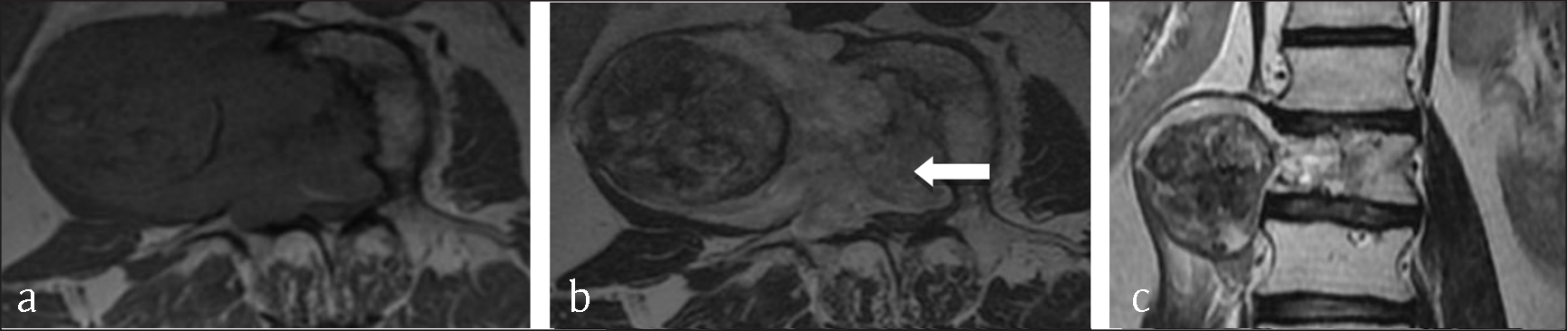
**Chordoma of L2.** MR. **(a)** Axial T1-weighted, **(b)** axial T1-WI after gadolinium contrast injection, coronal T2-WI **(c)** show a lobulated mass lesion of heterogeneous signal originating from the vertebral body of L2 with large soft tissue component extending into the paravertebral muscles (curved arrow) and anterior epidural space (arrow).

Chordomas show signal characteristics parallel to nucleus pulposus of the disk (notochord) on MR being low to intermediate signal on T1-WI (Figure 5a) and very high signal on T2-WI [19]. Coronal images on MRI demonstrate a “mushroom” appearance of the tumor (Figure 5c). High signal intensity on T1-WI may be seen and due to focal areas of hemorrhage and high protein content of the myxoid and mucinous components. The fibrous septa that divide the gelatinous components of the tumor are seen as areas of low signal intensity on T2-WI [1]. The presence of hemosiderin also accounts for the low signal intensity seen on T2-WI. After injection of gadolinium contrast, most chordomas show moderate heterogeneous enhancement (Figure 5b). Thick peripheral and septal enhancement similar to chondrosarcoma may be seen [20].

Chordoma should be differentiated from giant embryonic remnants of the notochord (disappearing during the second month of embryonal life). Unlike in chordoma, radiographs and CT fail to demonstrate a distinct lesion in giant notochordal rest, instead showing either normal bone or a variable degree of sclerosis [1]. Bone scintigraphy is typically normal, whereas MR shows a lesion of low T1- and high T2-WI signal intensity and no soft-tissue involvement. In doubtful cases, repeated CT scan or MRI imaging studies help ensure that the lesion is not progressive. When the tumor involves the sacral bone, the main differential diagnoses are chondrosarcoma and metastasis [21]. The purely lytic lesions may mimic plasmocytoma. Osteomyelitis and lymphoma should also be considered.

Chordoma is a low-grade and slowly growing tumor but generally has a poor long-term prognosis. Metasases are rare, estimated between 4 to 43% of patients [8], but local recurrence is frequent.

## Ewing Sarcoma and PNET

Ewing sarcoma and PNET are defined as round cell sarcomas that show varying degrees of neuroectodermal differentiation [22]. Ewing sarcoma is the most common nonlymphoproliferative primary malignant tumors of the spine in children and adolescents. The peak of incidence is usually seen in the second decade of life (mean age, 19.3 years), with a slight male predominance (62% vs 38% of cases) [1]. Lesions of the spine account for 3–10% of all primary sites of Ewing sarcoma [1]. The most common location is the sacrococcygeal region, followed by the lumbar and the thoracic spine. Cervical spine involvement is rare (3.2% of patients) [19]. In the nonsacral spine, most lesions (60%) originate in the posterior elements with extension into the vertebral body [1]. More than one segment is involved in 8% of cases. The disk spaces are usually preserved [23].

**Figure 6:**
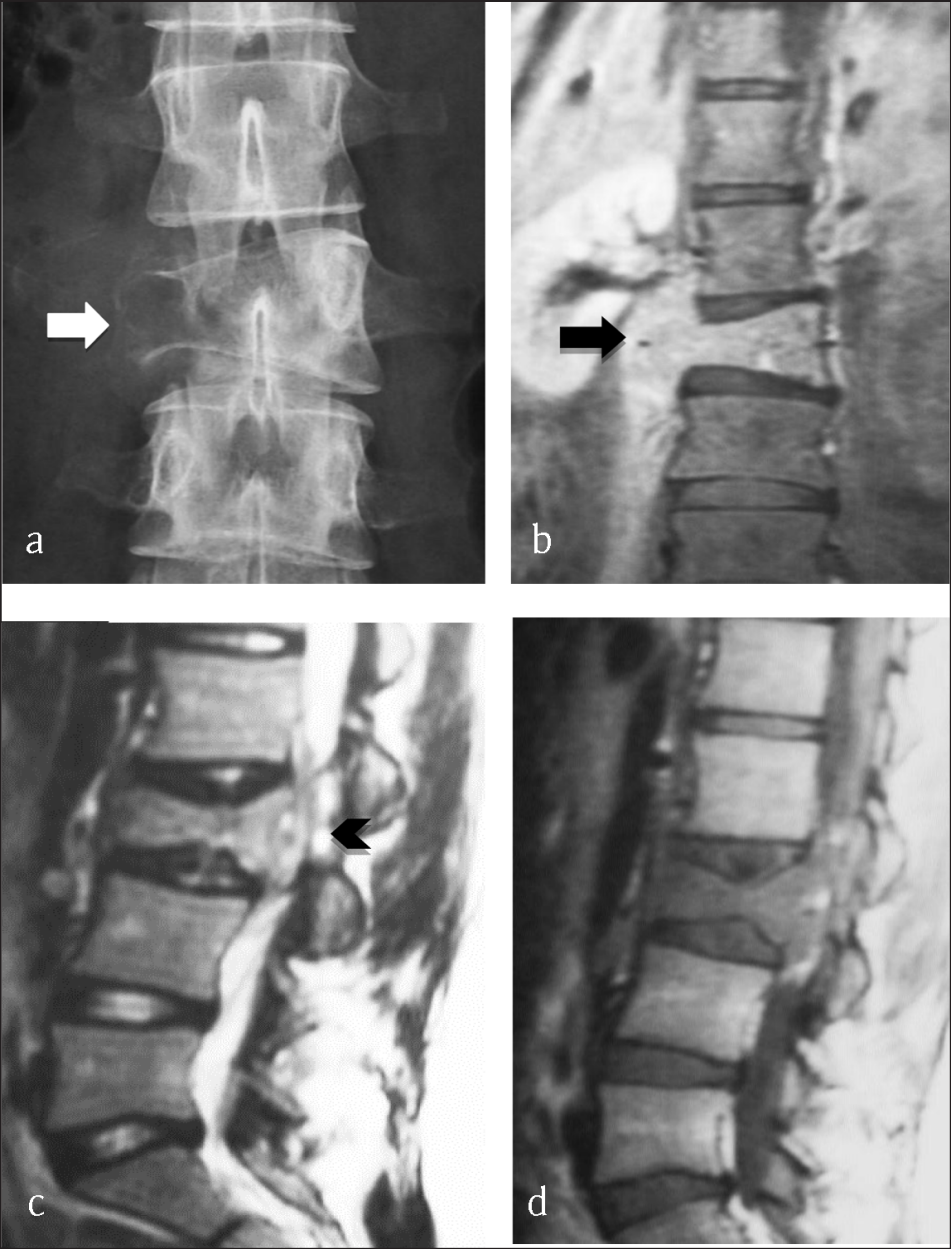
**Ewing Sarcoma of L3. (a)** Plain radiograph of lumbar spine reveals lytic lesion of right pedicle and right transverse process with partial vertebral collapse (arrow). **(b, c, d)** MR: coronal T2-WI, sagittal T2-WI and T1-WI after gadolinium reveal vertebral lesion of L3 with high signal intensity on T2-WI, enhancing after gadolinium injection and extending to prevertebral muscles (arrowhead) and to epidural space (arrow).

Pain is the most common symptom of patients suffering from spinal Ewing’s sarcoma [24]. Neurological deficit or radiculopathy is seen in 80% of patients [23]. Histologically, these tumors are composed of small round blue cells with large irregular sheets of cells divided by strands of fibrous tissue [25]. Several studies have confirmed a characteristic 11–22 chromosomal translocation in Ewing’s sarcoma in 85% of cases [26]. Lesions may be lytic (Figure 6a), sclerotic, or mixed. Almost all tumors (93%) are lytic and morphologically aggressive with large paraspinal soft tissue components that are usually larger than the intraosseous lesion. A purely sclerotic pattern is uncommon and may correspond to necrotic and reactive bone formation. Other unusual imaging findings include vertebra plana, ivory vertebra, and pseudohemangioma [27]. Invasion of the spinal canal is common (91% of cases) (Figure 6b and 6c).

The prognosis in Ewing sarcoma/PNET has improved in the modern era of treatment and current survival rate is estimated to be 41% [22]. The best therapeutic results are provided in radical resection and (neo-) adjuvant chemotherapy combined with radiation therapy [23].

## Lymphoma

Lymphoma is categorized as primary osseous lymphoma if bone is the only known site of disease for six months [28]. It is a rare extranodal manifestation of non-Hodgkin lymphoma, accounting for only about 1%–3% of all lymphomas and 7% of primary bone tumors [29]. Patients are between 40 to 60 years old with an equal male to female ratio [23]. In most cases, lymphoma involves the anterior vertebral column, whereas posterior involvement is rare.

Histologically, most bony lymphomas show a mixed cell infiltrate in which the cellular size and shape differ considerably. The very characteristic infiltrative pattern should suggest the diagnosis of lymphoma. The medullary bony trabeculae may show reactive sclerosis [23].

**Figure 7:**
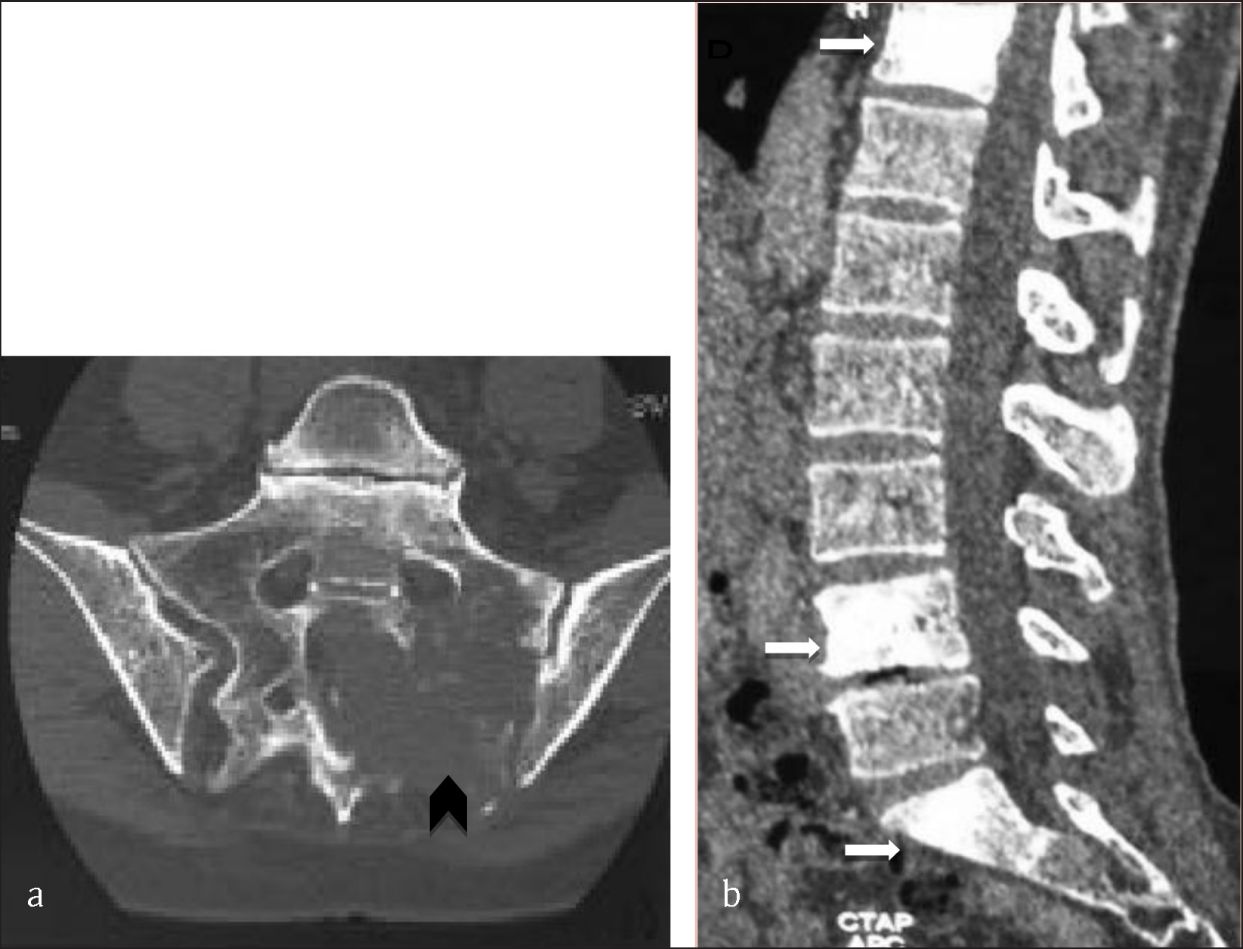
**Lymphoma of the sacrum.** Axial CT scan image shows a destructive lesion of sacrum extending through the left sacroiliac joint (arrowhead).

**Figure 8:**
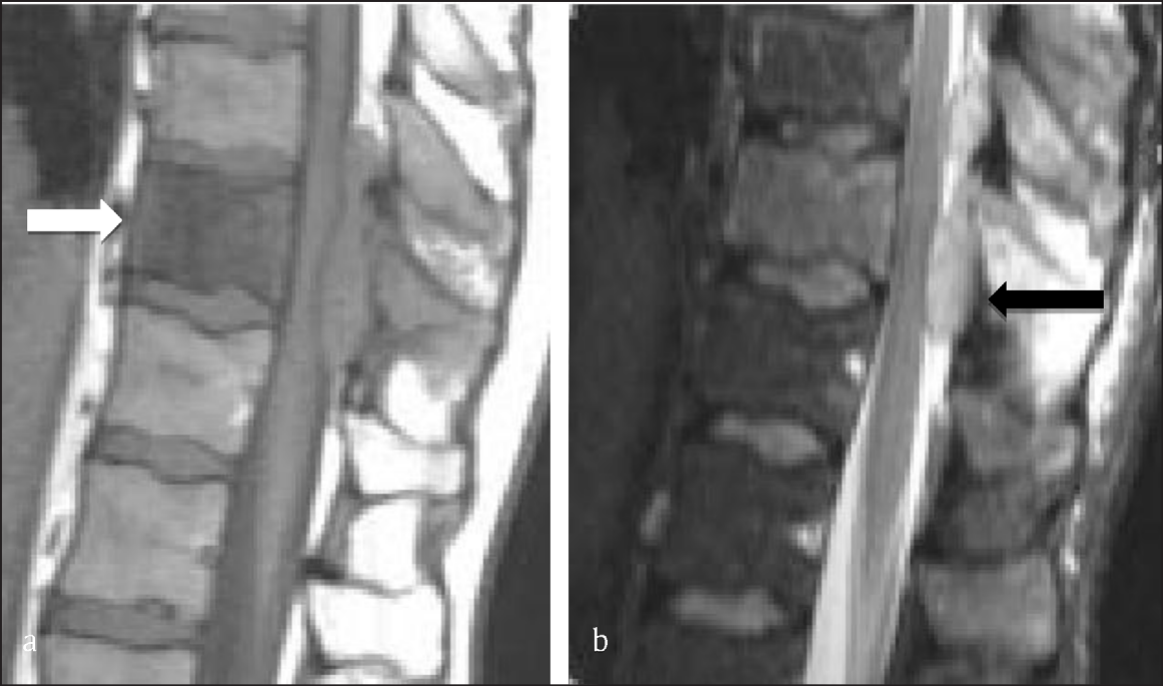
**B-cell lymphoma of L2.** MR: **(a)** Sagittal T1- and **(b)** T2-WI image show an osseous lesion of L2 (white arrow) extending into spinous process of L1 and L2 and in the posterior epidural spaces (black arrow) but without extension through the intervertebral disk.

Radiologically, the lesions are lytic (Figure 7), mixed, or rarely sclerotic causing ivory vertebra. Lymphoma should be considered in the differential diagnosis of any sclerotic vertebral lesion. Bony sequestra may be seen as well. MR is helpful to detect bone marrow involvement, and large soft tissue component appearing as areas of low signal intensity on T1-WI (Figure 8a) and hyperintense on T2-WI (Figure 8b) and variable enhancement. MR may also show a pathological fracture. Patterns of focal/multifocal uptake of 18F-FDG on a background of diffuse uptake at FDG PET/CT should be considered positive for bone marrow involvement. On the other hand, a completely diffuse (nonfocal) pattern of 18F-FDG uptake is nonspecific and may represent hematopoietic response to cytokines, especially after onset of treatment [28].

The treatment options of the primary bone lymphomas are surgical, chemotherapy, and radiotherapy [30]. Isolated spinal lymphoma responds very well to chemotherapy and radiotherapy. Lesions causing compression or complicated by pathological fracture with neurological deficits may require surgical decompression and stabilization using an anterior approach primarily followed by chemotherapy and radiation therapy.

## Plasmocytoma

**Figure 9:**
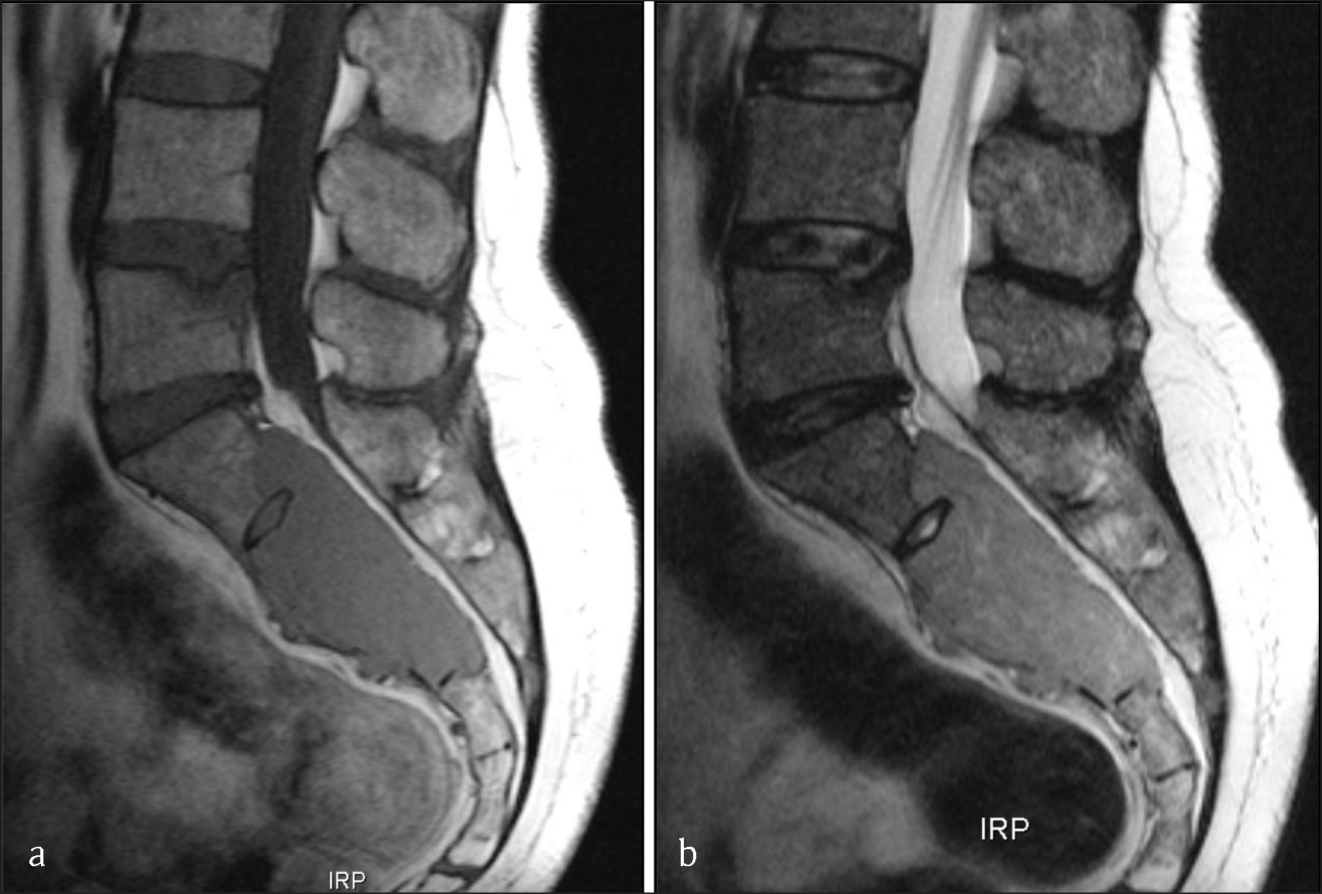
**Sacral plasmocytoma.** MR: **(a)** Sagittal T1-WI shows homogenous mass involving S1, S2 and S3 with intermediate signal intensity. **(b)** Sagittal T2-WI show a high signal intensity of the mass extending into the pelvis, epidural space, and posterior elements.

Plasmocytoma (Figure 9) is a clinical variant considered to represent an early stage of multiple myeloma, with stages of transition existing between the localized and the disseminated types [31]. It is an uncommon tumor occuring in 3–7% of patients with plasma cell neoplasms. Seventy percent of patients are over 60 years old [1], but it has also been reported in adolescents [32]. A monoclonal immunoglobulin is present at a low seric level in 40% of cases [1].

The most common symptom is local pain in the region of the affected spine, radicular pain or neurological deficit.

The spine is involved in 25–50% of the cases. Thoracic vertebrae are most commonly involved [33]. The male to female ratio is 2 to 3/1 [23]. The vertebral body is the most common site of involvement by plasmocytoma due to its rich red marrow content, but the tumor frequently extends to the pedicles [1]. Plain radiographs show a solitary radiolucent lesion within the axial skeleton or a high loss of vertebral body. Technetium bone scan is normal, except in case of a pathological fracture.

The treatment of choice is chemotherapy and/or radiation therapy at 40 Gy to 50 Gy [35]. Solitary plasmocytoma usually progresses to multiple myeloma in three to five years [34]. Surgery is performed for decompression and stabilization of the spine in case of pathological fracture, kyphosis, neurological deficit, or pain caused by instability [23].

## Multiple Myeloma

In 2014, the International Myeloma Working Group (IMWG) revised diagnostic criteria for multiple myeloma (MM), allowing the use of specific biomarkers and modern imaging tools to define the disease in addition to the established CRAB features [35]. Multiple myeloma is the third most common blood cancer in adults, with a reported prevalence of 4.3 cases per 100,000 population [36].

**Figure 10:**
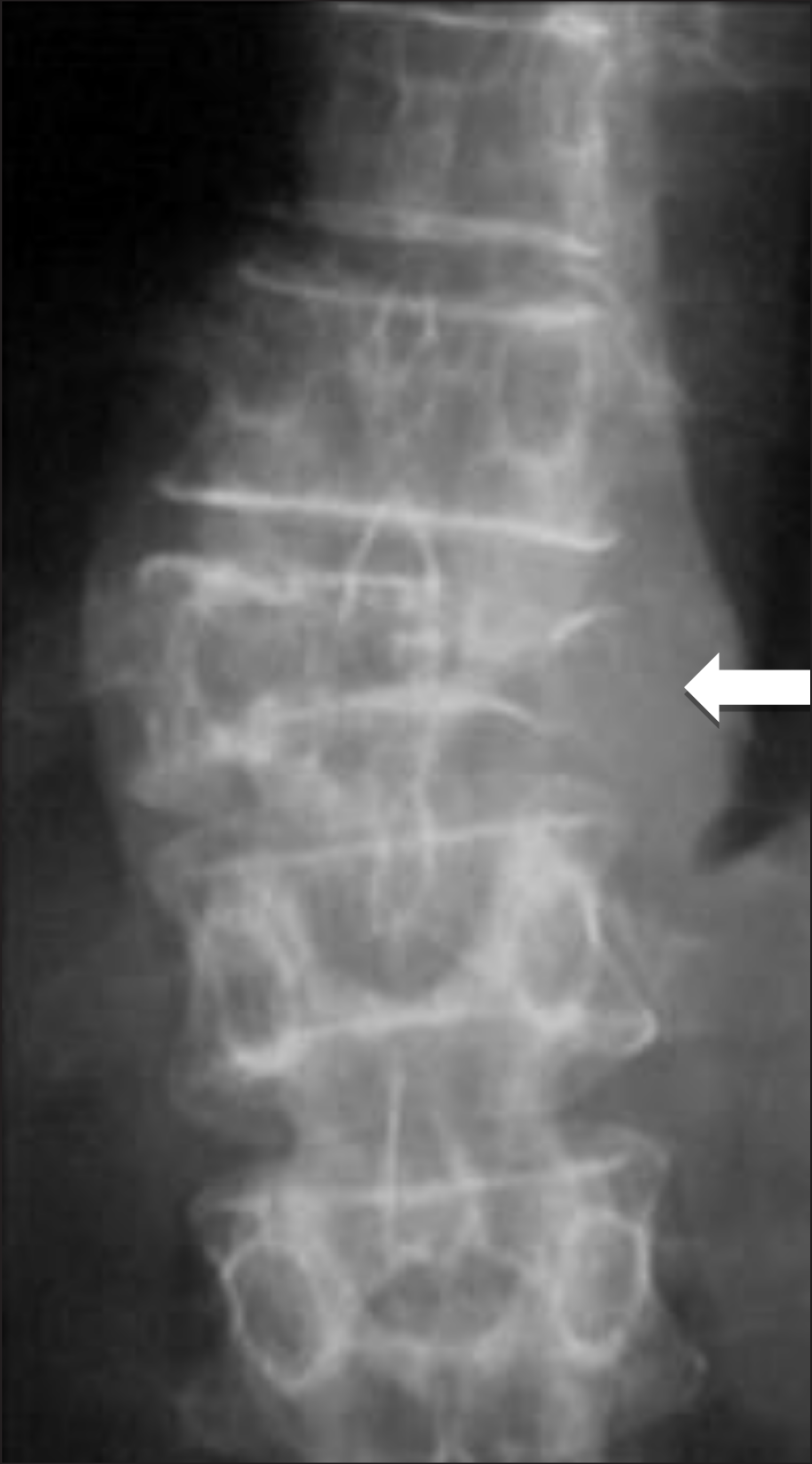
**Multiple myeloma.** Plain radiograph of spine shows an osteolytic lesion with cortical breakthrough, vertebral collapse and soft tissue mass (arrow).

Skeletal involvement occurs in 80–90% of patients with MM [37]. Vertebral involvement is observed in 65% of cases [38]. Multiple myeloma can affect any bone, including the skull, spine, pelvis, ribs, and proximal long bones. Therefore, a complete skeletal survey should include a postero-anterior and lateral view of the skull, spine, humeri, and femora, as well as an antero-posterior view of the pelvis and chest [39]. Radiographic findings of multiple myeloma include focal well-circumscribed lytic lesions without surrounding reactive sclerosis (70% of cases) [38] (Figure 10) or diffuse osteolysis [40]. Sclerotic lesions are less common and should raise the suspicion of POEMS syndrome (Polyneuropathy, Organomegaly, Endocrinopathy, M-protein and Skin changes) [41].

Ten to 20% of patients with multiple myeloma are normal on radiography [42,43]. Pathological compression fractures of the vertebral bodies related to osteopaenia and/or lytic lesions (Figure 10) are common. Bone scintigraphy is not useful for the assessment of therapy.

Although new or enlarging lesions generally mean disease progression, lytic bone lesions rarely show evidence of healing on plain radiographs, and routine follow-up skeletal survey is of no benefit [44].

Whole-body multidetector computerized tomography (WB-MDCT) is a preferable imaging tool over whole-body X-ray (WBXR) surveys for assessment of fracture risk and characterization of compression fractures [45]. Limitations of WBCT are a higher radiation [39], a limited evaluation of intramedullary bone invasion without cortical bone involvement leading to understaging, lack of specificity in evaluating osteopenia [46], and inability to assess response to therapy. Similar to WBXR, osseous lesions can remain unchanged on WBCT even after complete remission.

**Figure 11:**
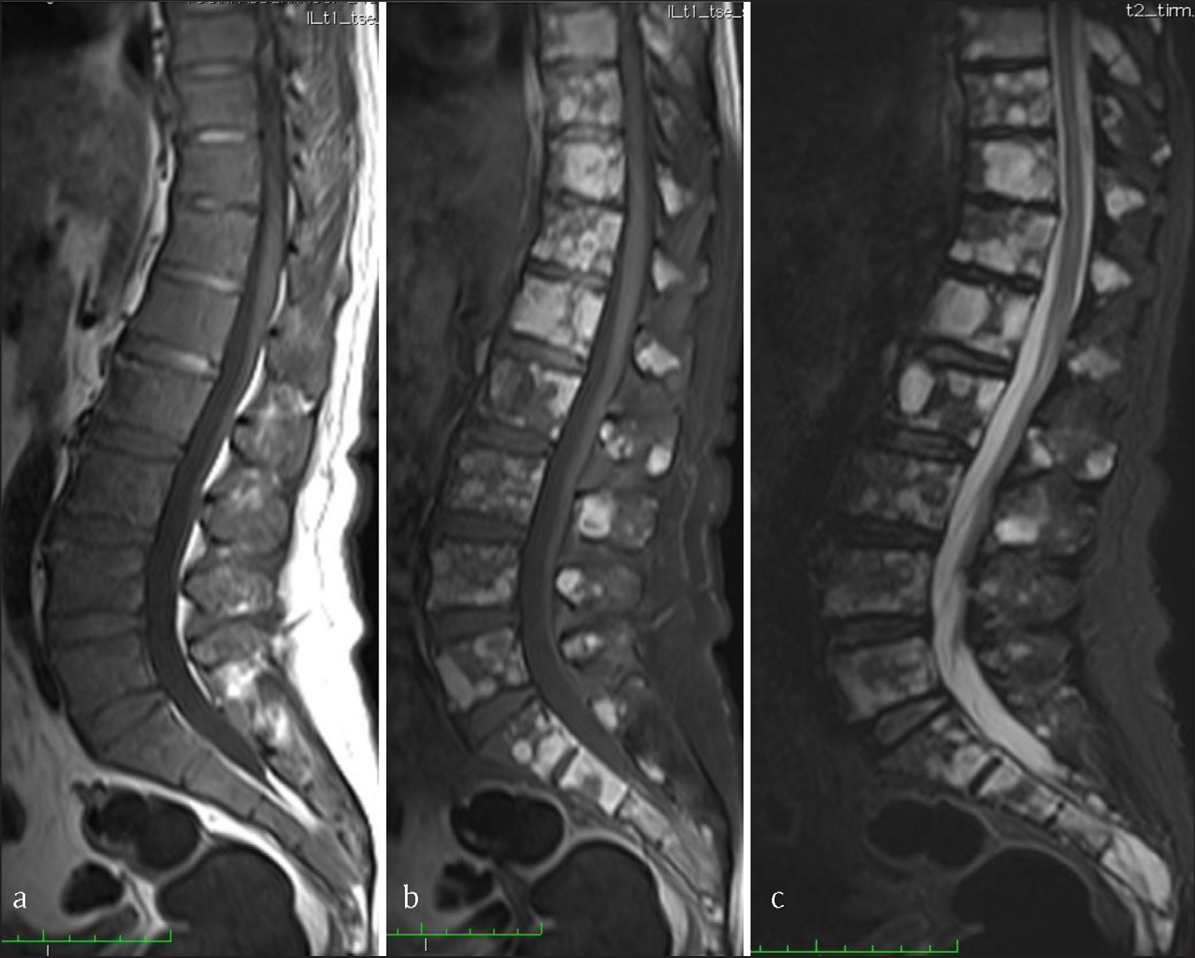
**Multiple myeloma.** MR: **(a)** Sagittal T1-WI, **(b)** sagittal fat-suppressed T1-WI after gadolinium contrast administration and **(c)** sagittal fat-suppressed T2-WI of the thoracolumbar spine display a diffuse bone marrow infiltration of vertebrae with low signal intensity on T1-WI and intermediate to high signal intensity on T2-WI. There is multifocal enhancement.

MR in patients with clinical and laboratory findings of symptomatic myeloma provides prognostic information and may detect unsuspected myeloma lesions [44]. Five different infiltration patterns can be differentiated on MR: normal appearance of bone marrow despite minor microscopic plasma cell infiltration, focal involvement, homogeneous diffuse infiltration, combined diffuse and focal infiltration, and “salt-and-pepper” pattern with inhomogeneous bone marrow with interposition of fat islands [47]. Typical MM lesion is hypointense on T1-WI (Figure 11a) and of moderate increased signal on T2-WI s (Figure 11c). However, due to the high signal intensity of normal bone marrow on T2-WI, fat suppression is required for a more sensitive detection rate. Contrast administration is not necessary routine, but shows enhancement of the lesion (Figure 11b); an enhancement greater than 40% is considered pathologic [48].

Abnormal signal can also be observed after chemotherapy and the use of growth factors or in young individuals with hyperplasia of normal haematopoiesis. Thus, the clinical context should be taken into account when evaluating MR, and MR should not be performed close to chemotherapy. Diffuse marrow infiltration has a decreased signal on T1-WI and an increased signal on STIR. The overall performance of MR is enhanced by applying dynamic contrast-enhanced MR and diffusion-weighted imaging sequences, providing additional functional information on bone marrow vascularization and cellularity [48].

Posttreatment MRI typically demonstrates a gradual replacement of marrow infiltration or focal lesions by marrow fat, but this may be delayed up to four or five years in some focal lesions [44].

FDG PET/CT is particularly sensitive for the detection of extramedullary disease and can help detect the metabolically active lesions that often precede evidence of osseous destruction at conventional radiography [49]. Both MR and FDG PET/CT allow accurate localization of disease after chemotherapy or autologous stem cell transplantation and can provide important prognostic information that can influence further clinical decision making regarding therapy, particularly when tumor serum markers may be a less reliable indicator of disease burden after repeated treatment [49].
